# Correction to “How Do Open‐Ended Interview and Closed‐Ended Questionnaire Responses Compare? A Matched Mixed‐Methods Study Examining Treatment Attitudes of Patients With Meniscal Tear and Persistent Pain”

**DOI:** 10.1002/acr2.90149

**Published:** 2026-09-06

Oh, P.M. , Fox, K.B. , Dhani, J. , Carland, A.L. , Long, K. , Selzer, F. , Katz, J.N. , Bisson, L.J. and Weiss‐Laxer, N.S. (2026), How Do Open‐Ended Interview and Closed‐Ended Questionnaire Responses Compare? A Matched Mixed‐Methods Study Examining Treatment Attitudes of Patients With Meniscal Tear and Persistent Pain. ACR Open Rheumatology, 8: e90029. 10.1002/acr2.90029 41947721 PMC13058571

In figure 2, the joint displays are missing figure keys describing what the colors represent. This should have included the figure keys.


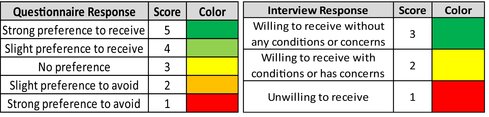


We apologize for this error.

